# Randomized immunotherapy trial in dual‐allergic patients using “active allergen placebo” as control

**DOI:** 10.1111/all.13842

**Published:** 2019-05-28

**Authors:** Martin Wagenmann, Margitta Worm, Yasemin Akboga, Martin Karjalainen, Jens M. Hohlfeld

**Affiliations:** ^1^ Department of Otorhinolaryngology Düsseldorf University Hospital (UKD) Düsseldorf Germany; ^2^ Division of Allergy and Immunology Department of Dermatology, Venereology and Allergy Charité‐Universitätsmedizin Berlin Berlin Germany; ^3^ Department of Research and Clinical Development Allergopharma GmbH & Co. KG, A Business of Merck Reinbek Germany; ^4^ Fraunhofer Institute for Toxicology and Experimental Medicine Department of Respiratory Medicine Hannover Medical School German Center for Lung Research Hannover Germany

**Keywords:** active allergen placebo, active control group, allergen challenge chamber, allergen immunotherapy, dual allergy

## Abstract

**Background:**

Placebo control in allergen immunotherapy (AIT) trials presents ethical and blinding concerns. We tested a trial design with an “active allergen placebo,” as proposed by ARIA‐GA ^2^ LEN, to investigate in a double‐blind trial the efficacy and safety of AIT in dual‐allergic patients (grass and birch pollen) using active untargeted treatments as controls.

**Methods:**

We randomized 95 patients to receive either grass (N = 47) or birch AIT (N = 48). Patients were exposed to both allergens in an allergen challenge chamber (ACC) before and after 9 months of AIT. Targeted (ACC‐allergen = AIT‐allergen) and untargeted (ACC‐allergen ≠ AIT‐allergen) treatment effects were assessed.

**Results:**

Immunotherapy reduced significantly the mean (95% confidence interval) area under the curve of total nasal symptom score (targeted effects) by −13.55 (−17.56, −9.54; *P* < 0.001) after grass and −9.81 (−14.13, −5.50; *P* < 0.001) after birch AIT. Differences in targeted vs untargeted effects between AIT groups (utility of control group) were statistically significant for both grass (*P* = 0.02) and birch (*P* = 0.02) allergens. Targeted vs untargeted differences within‐treatment groups (specificity of ACC measurement) were significant for grass AIT (*P* < 0.001) but not significant for birch AIT group (*P* = 0.24). Specific immunoglobulin G_4_ to both allergens increased significantly (*P* < 0.001) after targeted treatment, while remained unchanged for untargeted treatments. Both treatments were well tolerated.

**Conclusions:**

Immunotherapies for both grass and birch allergens were efficacious and safe. The study confirms the specificity of AIT. Untargeted treatment groups could serve as controls in future AIT trials.

## 1 INTRODUCTION

Placebo control in allergen immunotherapy (AIT) trials poses methodological and ethical challenges. Fewer local side effects in the placebo arm than in the active arm may impact blinding.^1^ Ethical considerations, especially in pediatric populations, are also present since not all patients can benefit from active therapy during long trials.^2^

ARIA‐GA^2^LEN statement (2011) introduced the theoretical concept of an “active allergen placebo,” that is, a placebo with the other season's allergen. In this study design concept, patients allergic to 2 different allergens are randomized to AIT with 1 of 2 allergens, with the untargeted treatment serving as control for the targeted treatment.^1^ Targeted treatment effect in this context is defined as the AIT effect directed at 1 of the 2 patient's allergies (eg, grass AIT effect on the grass allergy—direct or expected effect), while the untargeted treatment effect is the effect on the other allergy (eg, grass AIT effect on the birch allergy—indirect or unexpected effect). In this proposed design, all patients would benefit from active treatment. This trial design was used previously in a post hoc analysis of a study involving few patients suffering from house dust mite and timothy grass pollen allergy.^3^ However, to further validate the trial design in AIT trials, there is a need to investigate how specific the AIT is and to what extent treatment effects are also present in the untargeted treatment groups. Also, it should be assessed whether such studies can be done in patients suffering from distinct pollen allergens.

To address these questions, we designed a prospective, double‐blind, clinical trial in which patients with dual grass and birch pollen allergy were randomized to either grass or birch AIT. Untargeted AIT served as a control for targeted AIT. The total nasal symptom score (TNSS) pre‐ and post‐treatment was evaluated for all patients during separate grass and birch pollen exposures in an allergen challenge chamber (ACC). The main objectives of this clinical trial were to demonstrate a treatment effect (post‐ vs pretreatment) for grass and birch targeted AIT and to compare the treatment effect between targeted and untargeted treatments. To our knowledge, this is the first trial investigating the treatment effects of AIT in targeted and untargeted treatments in dual‐allergic patients under validated conditions of an ACC.

## 2 METHODS

### 2.1 Trial design

This was a multicenter, randomized (1:1), double‐blind, controlled phase IV trial with 2‐active parallel groups, conducted in Germany in 15 investigational sites (10 hospital clinics and 5 office practices) between April 2014 and November 2015 (EudraCT: 2013‐003095‐12).

### 2.2 Participants

Eligibility criteria (all of which needed to be met for inclusion) were as follows: patients (male/female) aged 18‐65 years, suffering from immunoglobulin E (IgE)‐mediated seasonal allergic rhinoconjunctivitis, with or without controlled asthma, caused by grass *and* birch pollen allergy. Asthma control was documented according to the Global Initiative for Asthma (GINA) 2012. Allergy was documented by skin prick test wheal ≥ 3 mm in diameter; histamine wheal ≥ 3 mm; NaCl control reaction ≤ 2 mm; positive immunoassay result for specific IgE > 0.70 kU/L; main discomfort due to allergic rhinoconjunctivitis during grass and birch pollen seasons; and treatment with antiallergics for ≥ 2 years prior enrollment. Additionally, prior to randomization, patients had to show relevant symptoms (adjusted area under the curve [AUC_adj_] of TNSS ≥ 10 points) to both allergens at the 2‐hour pretreatment ACC exposure.

### 2.3 Interventions

**Figure 1:**
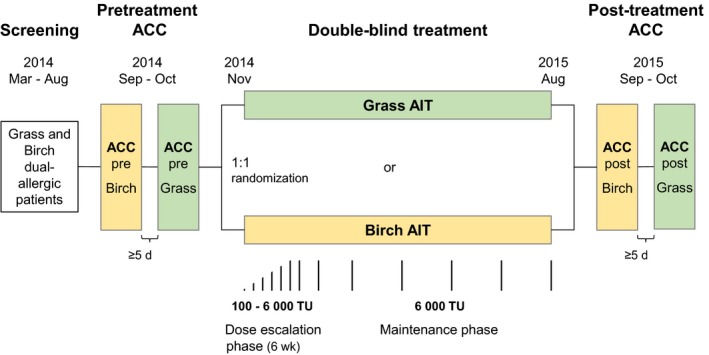
Trial design. ACC, allergen challenge chamber; AIT, allergen immunotherapy; TU, therapeutic units. Subcutaneous injections (upper arm) were administered first at dose escalation steps of 7 d (100, 200, 400, 800, 1500, 3000, and 6000 TU); the maintenance dose of 6000 TU was then administered after 2 wk, and then after 4 wk and finally extended to intervals of 6‐8 wk

At screening visit, a skin prick test (see Supporting Information for details), immunological profile assessment (see Supporting Information), and lung function test (peak expiratory flow [PEF]) were performed. Before randomization, 2 distinct pretreatment ACC visits (Figure 1) were performed for the 2 different allergens. Patients were then randomized to receive either grass or birch AIT. After the treatment, 2 additional distinct post‐treatment ACC visits were performed. At final visit, ≥ 5 days after the last ACC, the following outcomes were evaluated: adverse events (AEs), laboratory tests, concomitant medication, tolerability, PEF (safety measurement per investigator judgment), and specific immunoglobulin G_4_ (IgG_4_) timothy grass and birch titers.

### 2.4 Immunotherapy

Investigational Medicinal Products (IMPs) were 100% aluminum‐adsorbed allergoid preparations of ALLERGOVIT^®^ grass pollen (*Phleum pratense*,* Lolium perenne*,* Festuca pratensis*,* Holcus lanatus*,* Dactylis glomerata*, and *Poa pratensis*) or ALLERGOVIT^®^ birch pollen (100% *Betula verrucosa*) (Allergopharma GmbH & Co. KG). Both preparations were provided at strength A (1000 therapeutic units (TU)/mL) or B (10 000 TU/mL). Sponsor manufactured the IMPs according to the revised Good Manufacturing Practices Guidelines of the World Health Organization. Subcutaneous injections were administered first at dose escalation steps of 7 days (100, 200, 400, 800, 1500, 3000, and 6000 TU) until the maintenance dose of 6000 TU was reached (Figure 1). The maintenance dose was then administered after 2 weeks and then after 4 weeks and finally extended to intervals of 6‐8 weeks. Each patient was treated for approximately 9 months, including 1 grass and 1 birch pollen season, without reduction of the maintenance dose during the pollen seasons.

### 2.5 Allergen challenge chamber and TNSS

Allergic reactions were assessed at the ACC of the Fraunhofer Institute for Toxicology and Experimental Medicine (ITEM) (Hannover, Germany).^4^ Technical details are summarized in the Supporting Information. The challenge lasted 120 minutes, and patients were under constant medical supervision. *Betula* pendula and *Dactylis glomerata* were used as challenge agents (Allergon AB, Välingevägen 309, SE‐262 92 Ängelholm, Sweden). Both pre‐ and post‐treatment ACCs were performed ≥ 2 months outside the relevant pollen seasons.

The TNSS was the sum of scores for nasal congestion, rhinorrhea, nasal itching, and sneezing, using a 4‐point scale (0‐3).^5^ Each ACC visit included 1 pretreatment TNSS assessment prior to entering the challenge chamber and 6 assessments during allergen challenge (20, 40, 60, 80, 100, and 120 minutes). The differences between each TNSS assessment during allergen challenge and pretreatment were added to calculate the AUC_adj_ of the TNSS for every ACC exposure pre‐ and post‐treatment. AUC_adj_ of TNSS could range between −12 and 72; however, per inclusion criteria, patients were required to have a pretreatment AUC_adj_ of TNSS of ≥ 10 for both allergens.

### 2.6 Immunological parameters

Immunological AIT effects were assessed by analyzing the serum concentrations of timothy grass and birch‐specific IgG_4_ before and after treatment using ImmunoCAP (ThermoFisher Scientific).

### 2.7 Numerical rating scale (NRS)

The patients rated symptoms at home using an NRS (discrete values: 1[good] to 10[poor]) once during the birch (May 04‐10, 2015) and once during the grass pollen season (June 22‐28, 2015), while under treatment. Information regarding the extent of the natural grass and birch pollen exposition in Germany was obtained from http://www.pollenflug-nord.de/.

### 2.8 Outcomes

Treatment effect, on targeted and untargeted AIT groups, was defined for each treatment as the difference of the AUC_adj_ of TNSS between pre‐ and post‐treatment ACC measurements. Treatment effects for targeted treatments are defined as grass‐on‐grass (G‐G) treatment effect and birch‐on‐birch (B‐B) treatment effect and for untargeted treatments as birch‐on‐grass (B‐G) treatment effect and G‐B (grass‐on‐birch) treatment effect (Figure 3). In other words, the first letter of each abbreviation refers to the pollen species used in the ACC exposure and the second letter to the allergen preparation selected for AIT.

The primary efficacy outcomes were G‐G and B‐B treatment effects. Secondary efficacy outcomes were the treatment effect of targeted vs untargeted AITs:

- Between AIT groups, to assess utility of the “active allergen placebo”: G‐G vs G‐B; and B‐B vs B‐G;
- Within AIT groups, to assess specificity of the ACC measurement: G‐G vs B‐G; and B‐B vs G‐B;
- Combined: (G‐G & B‐B: average of both effects) vs (B‐G & G‐B).

The change from pre‐ to post‐treatment in allergen‐specific IgG_4_ was also investigated. Severity of allergy symptoms was measured during pollen seasons, and NRS scores were compared between birch and pollen seasons (within each AIT group). Safety outcomes included AEs, laboratory tests, vital signs, and assessment of overall tolerability by investigators and patients.

### 2.9 Safety measurements

Safety evaluation was based on AEs, lung function tests, laboratory tests, and physical examination. Tolerability was assessed by the investigator and patients on a 5‐point Likert scale.

### 2.10 Medications

Rescue medications were permitted for ACC related symptoms, which included salbutamol, topical levocabastine nasal spray and eye drops, and loratadine or cetirizine tablets. Established symptomatic medications during the pollen season were allowed. Intermittent treatment with inhaled corticosteroids was allowed at ≤ 500 μg/day beclomethasone‐dipropionate (or equivalent) for patients with asthma.

### 2.11 Randomization and blinding

To ensure a 1:1 randomization ratio within each trial site, the randomization was performed blockwise. Block sizes were unknown to trial sites. The investigator requested randomization numbers via electronic case report form. At screening visit, patients were assigned a 6‐digit screening number which identified the patient during the trial. At randomization visit, eligible patients were additionally assigned a 3‐digit random number in ascending order of inclusion within each trial site. The random list was not accessible to the trial team before trial end. IMPs and respective vials had identical appearance and trial sites received IMPs in a blinded fashion.

Patients were assigned to the following analysis sets: *safety set (SAF)*, patients who received ≥ 1 IMP dose; *full analysis set (FAS)*, patients of the SAF for whom an efficacy assessment after randomization was available; and *per‐protocol set (PPS)*, patients of the FAS without major protocol violations. Allocation to analysis sets and decisions on protocol violations were made at the blind data review meeting prior to breaking the blind.

### 2.12 Statistical methods

The primary endpoint was analyzed for the FAS (confirmatory) and PPS (exploratory, sensitivity analysis). For the primary and secondary analyses, a hierarchical test procedure was applied. Since grass AIT data in an ACC were available,^6^ the hypothesis H_01_: (μ_G‐G_ = 0 vs μ_G‐G_ ≠ 0) was tested first at α = 0.05. If H_01_ could be rejected, H_02_ (μ_B‐B_ = 0 vs μ_B‐B_ ≠ 0) would be tested at α = 0.05. If H_01_ could not be rejected, the procedure stopped and H_02_ was not tested. H_01_ and H_02_ were performed with a 1‐sample *t* test for each AIT group separately. If H_01_ and H_02_ had been rejected, the secondary endpoints were tested 2‐sided at α = 0.05. All secondary efficacy and safety endpoints were analyzed descriptively for the FAS. Analyses were performed using Statistical Analysis Software (SAS) version 9.3 or higher. No relevant changes to the planned analyses were performed.

In a previous trial (AL1011av, EudraCT: 2011‐000674‐58), after a comparable treatment with grass AIT, a mean (±standard deviation) change in the area under the curve of the TNSS of −6.5 (±10.3) was observed. We assumed that the change in patients treated with birch was not < −5.0 with a comparable standard deviation. The power for rejecting H_01_ and H_02_ at a 2‐sided local alpha level of 0.05 was ≥ 90%, each, if 50 patients per AIT group could be analyzed. To account for a dropout rate of approximately 15%, 60 patients per AIT group were planned to be randomized.

### 2.13 Ethical conduct of the trial

The trial was conducted in accordance with the ethical principles (Declaration of Helsinki, 2002), Good Clinical Practice (ICH E6(R1), 1996, Directive 2001/20/EC, 2001), and the applicable regulatory requirements. All patients gave informed written consent, and an independent ethics committee approved all relevant trial documents.

## 3 RESULTS

**Figure 2:**
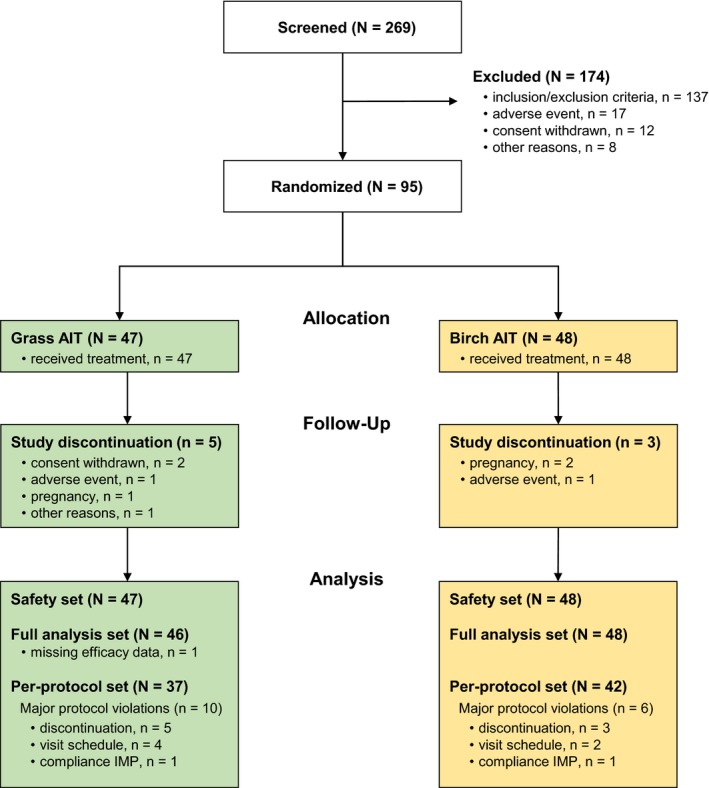
Patients’ flow diagram. AIT, allergen immunotherapy; N, number of patients; n, number of patients in group; IMP, Investigational Medicinal Product

Of the 269 patients screened, 95 were randomized to grass AIT (N = 47) or birch AIT (N = 48) (Figure 2). Of the 137 patients excluded due to inclusion/exclusion criteria, 28 patients had low symptoms (AUC_adj_ TNSS < 10) in the pretreatment ACC (4 patients during grass ACC and 24 patients during birch ACC). AIT groups were well balanced in demographic characteristics, and in total and specific IgE (Table 1). The majority of patients (n = 68; 71.6%) had no asthma reported, and about one‐third (n = 27; 28.4%) had controlled asthma. Controlled asthma was reported by 21.3% of patients on grass AIT and 35.4% on birch AIT. The mean (95% CI) number of injections (grass AIT: 12.8 [12.2, 13.5] injections; birch AIT: 12.8 [12.4, 13.1] injections) and treatment duration (grass AIT: 245.7 [230.7, 260.6] days; birch AIT: 251.0 [239.5, 262.5] days) was similar between AIT groups. All but 1 patient (grass AIT) reached the planned maintenance dose.

**Table 1:** Baseline demographic characteristics (safety set)

	Grass AIT (N = 47)	Birch AIT (N = 48)	Overall (N = 95)
Mean age (SD)	34.2 (9.8)	33.0 (11.2)	33.6 (10.5)
Sex, n (%)			
Female	25 (53.2%)	28 (58.3%)	53 (55.8%)
Male	22 (46.8%)	20 (41.7%)	42 (44.2%)
Mean BMI (SD)	24.86 (4.53)	24.95 (4.33)	24.91 (4.41)
Ethnic group, n (%)			
Caucasian	42 (89.4%)	45 (93.8%)	87 (91.6%)
Asian descent	3 (6.4%)	1 (2.1%)	4 (4.2%)
Other	2 (4.3%)	2 (4.2%)	4 (4.2%)
Smoking status, n (%)			
Nonsmoker	36 (76.6%)	32 (66.7%)	68 (71.6%)
Ex‐smoker	5 (10.6%)	8 (16.7%)	13 (13.7%)
Current smoker	6 (12.8%)	8 (16.7%)	14 (14.7%)
Household pets, n (%)			
No pets	39 (83.0%)	38 (79.2%)	77 (81.1%)
At present	5 (10.6%)	8 (16.7%)	13 (13.7%)
Formerly	3 (6.4%)	2 (4.2%)	5 (5.3%)
Allergic symptoms history, n (%)			
Nose symptoms	47 (100.0%)	48 (100.0%)	95 (100.0%)
Eye symptoms	47 (100.0%)	46 (95.8%)	93 (97.9%)
Lung symptoms	10 (21.3%)	16 (33.3%)	26 (27.4%)
Asthma control, GINA guidelines (2012), n (%)			
Controlled asthma	10 (21.3%)	17 (35.4%)	27 (28.4%)
Uncontrolled asthma	0	0	0
No asthma	37 (78.7%)	31 (64.6%)	68 (71.6%)
Other allergic diseases, n (%)	21 (44.7%)	27 (56.3%)	48 (50.5%)
Skin prick test, mean longest diameter of wheel in mm (SD)			
Negative control	0.0 (0.15)	0.0 (0.14)	0.0 (0.14)
Positive control	6.2 (1.65)	6.3 (1.34)	6.3 (1.49)
6‐grasses mix	10.9 (4.14)	10.0 (3.44)	10.4 (3.81)
Birch	11.2 (3.97)	10.4 (3.35)	10.8 (3.67)
Mean total and specific IgE in IU/mL (SD)			
Total	403.0 (622.77)	423.8 (1049.51)	413.5 (860.61)
Grass mix	35.5 (36.35)	31.4 (36.12)	33.5 (36.10)
Birch	34.4 (34.54)	24.3 (24.70)	29.3 (30.24)

Abbreviations: AIT, allergen immunotherapy; BMI, body mass index; GINA, Global Initiative for Asthma; IgE, immunoglobulin E; N, number of patients; n, number of patients with data; SD, standard deviation.

### 3.1 Primary endpoint (targeted treatment effects)

**Figure 3:**
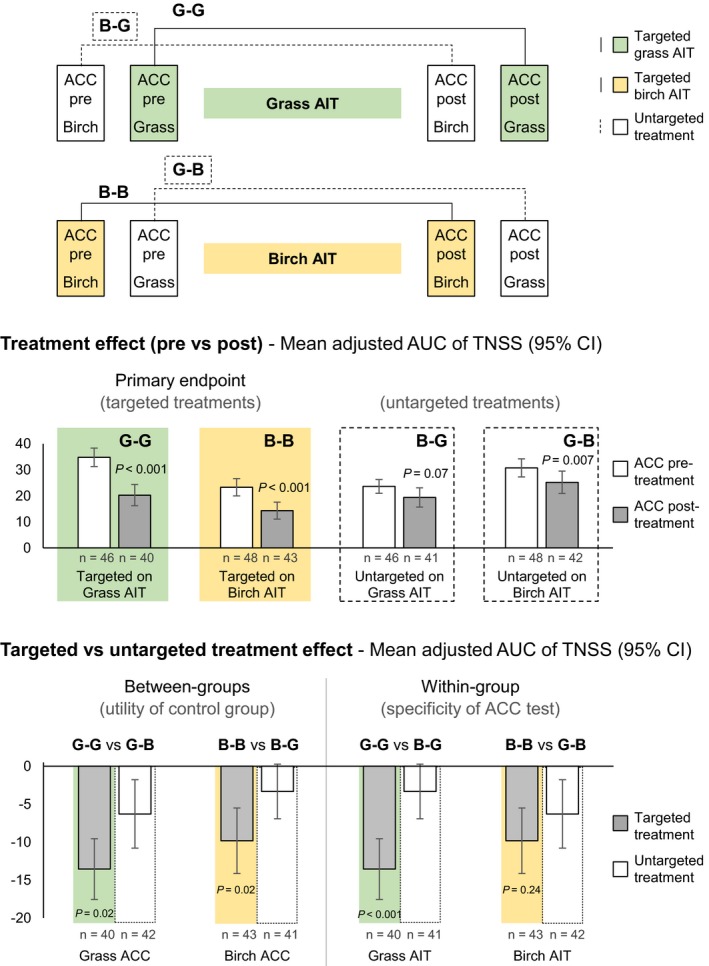
Overview of targeted and untargeted effects of allergen immunotherapy on total nasal symptom score (full analysis set). ACC, allergen challenge chamber; AIT, allergen immunotherapy; AUC, area under the curve; B‐B, birch‐on‐birch treatment effect (targeted); B‐G, birch‐on‐grass treatment effect (untargeted); CI, confidence interval; G‐B, grass‐on‐birch treatment effect (untargeted); G‐G, grass‐on‐grass treatment effect (targeted); n, number of patients with data; TNSS, total nasal symptom score. Between‐groups comparisons evaluate the utility of an “active allergen placebo”; within‐group comparisons evaluate the specificity of the ACC measurement. *P*‐value from 2‐sided 1‐sample *t* test. Results for the per‐protocol set analysis were similar to full analysis set for all primary and secondary endpoints

Patients treated with grass AIT (G‐G) had a statistically significantly lower mean (95% CI) AUC_adj_ TNSS post‐treatment (20.28 [16.20, 24.35]) than at pretreatment (34.80 [31.25, 38.36]), with a difference of −13.55 (−17.56, −9.54; *P* < 0.001) (Figure 3 G‐G), corresponding to a symptom reduction of 38.9%. Patients treated with birch AIT (B‐B) had also a lower mean (95% CI) AUC_adj_ TNSS post‐treatment (14.35 [11.09, 17.61]) than at pretreatment (23.31 [19.99, 26.64]), with a difference of −9.81 (−14.13, −5.50; *P* < 0.001) (Figure 3 B‐B), corresponding to a symptom reduction of 42.1%.

### 3.2 Secondary endpoints

#### 3.2.1 Targeted vs untargeted treatment effects

Patients treated with grass AIT and exposed to birch pollen in the ACC showed a slight reduction in mean (95% CI) AUC_adj_ TNSS of −3.32 (−6.92, 0.29), which was not statistically significant (*P* = 0.07) and corresponded to a symptom decrease of 14.0% (Figure 3 B‐G, untargeted). Patients treated with birch AIT and exposed to grass pollen showed a more pronounced reduction in mean AUC_adj_ TNSS of −6.29 (−10.79, −1.78), which was statistically significant (*P* = 0.007) and corresponded to a symptom decrease of 20.5% (Figure 3 G‐B, untargeted).

Between AIT groups (ie, comparing the same allergens challenges in different AIT groups), the targeted treatment effect was statistically significantly larger than the untargeted treatment effect for both grass (G‐G vs G‐B, *P* = 0.02) and birch (B‐B vs B‐G, *P* = 0.02) allergens (Table 2 and Figure 3). Within AIT groups (ie, comparing different allergens challenges in the same AIT group), the targeted treatment effect was statistically significantly larger than the untargeted effect for grass AIT (G‐G vs B‐G, *P* < 0.001), but not significant for birch AIT (B‐B vs G‐B, *P* = 0.24). The mean (95% CI) combined targeted (G‐G & B‐B, N = 83) treatment effect was −11.61 (−14.54, −8.69), which was statistically significantly larger (*P* < 0.001) than the combined untargeted (B‐G & G‐B, N = 83) treatment effect of −4.82 (−7.67, −1.97).

**Table 2:** Targeted and untargeted treatment effects on grass and birch AIT groups on the total nasal symptom score (full analysis set)

	Treatment effect (ACC post‐treatment – ACC pretreatment), adjusted AUC of TNSS			
	Grass AIT (N = 46)		Birch AIT (N = 48)	
	Targeted treatment postgrass –pregrass	Untargeted treatment postbirch – prebirch	Targeted treatment postbirch – prebirch	Untargeted treatment postgrass – pregrass
n (missing)	40 (6)	41 (5)	43 (5)	42 (6)
Mean (SD)	−13.55 (12.55)	−3.32 (11.42)	−9.81 (14.01)	−6.29 (14.46)
95% CI (Mean)	−17.56, −9.54	−6.92, 0.29	−14.13, −5.50	−10.79, −1.78
Median	−12.5	−5.0	−7.0	−6.0
Min, Max	−38.0, 18.0	−25.0, 22.0	−70.0, 13.0	−43.0, 31.0
Q25, Q75	−23.0, −7.0	−10.0, 3.0	−17.0, −1.0	−15.0, 4.0
Within‐group comparison Targeted vs Untargeted				
*P*‐value*	< 0.001		0.24	
Between‐group comparison Targeted vs Untargeted for ACC grass : [postgrass – pregrass] on grass treatment vs [postgrass – pregrass] on birch treatment				
*P*‐value*	0.02			
Between‐group comparison Targeted vs Untargeted for ACC birch : [postbirch – prebirch] on birch treatment vs [postbirch – prebirch] on grass treatment				
*P*‐value*	0.02			

Abbreviations: ACC, allergen challenge chamber; AIT, allergen immunotherapy; AUC, area under the curve; CI, confidence interval; Max, maximum; Min, minimum; N, number of patients; n, number of patients with data; Q, quartile; SD, standard deviation; TNSS, total nasal symptom score.

*P‐value from 2‐sided 1‐sample *t* test.

#### 3.2.2 Change in birch/timothy grass IgG_4_

Median (25th‐75th) serum levels of grass pollen‐specific IgG_4_ were higher postgrass AIT (1.96 [0.8‐4.0] mg/L) than at pretreatment (0.23 [0.2‐0.4] mg/L) (*P* < 0.001, 2‐sided Wilcoxon signed rank test). Similarly, median serum levels of birch pollen‐specific IgG_4_ were higher postbirch AIT (2.12 [1.1‐3.5] mg/L) than at pretreatment (0.42 [0.2‐0.9] mg/L) (*P* < 0.001). No differences between post‐ and pretreatment values were observed for grass and birch pollen‐specific IgG_4_ values for untargeted AITs (Figure S1).

#### 3.2.3 Severity of allergy symptoms during pollen seasons

Patients treated with grass AIT had a slightly higher median (25th‐75th) NRS score (4.0 [3.0‐6.0]) in the birch pollen season than in the grass pollen season (3.0 [2.0‐6.5]), which was not statistically significant (*P* = 0.52, 2‐sided Wilcoxon signed rank test). Similarly, patients treated with birch AIT had slightly higher median NRS score in the grass pollen season (5.0 [3.0‐7.0]) than in the birch pollen season (4.0 [3.0‐5.0]), which was also not statistically significant (*P* = 0.08).

#### 3.2.4 Safety results

The majority of patients reported mild (37.9%) or moderate (31.6%) treatment‐emergent AEs (TEAEs); 4 patients (4.2%) reported ≥ 1 severe TEAE. Most frequently reported TEAEs were nasopharyngitis (29.5% of 95 patients) and injection site swelling (16.8%), erythema (16.8%), and pruritus (14.7%). Vital signs at final visit did not show any clinically significant change from baseline. Tolerability results were similar in both AIT groups: assessed as “good” by 46.7% of patients and 50.5% of investigators and as “very good” by 38.0% of patients and 41.9% of investigators. AIT did not negatively impact lung function in patients with controlled asthma (Table S1).

Systemic and local reactions occurred with similar frequency between AIT groups (Table 3). TEAEs classified as systemic reactions (WAO^7^ grading) occurred in 3 patients: sneezing (2 patients, WAO Grade 1); and anaphylactic reaction with symptoms of dyspnea and angioedema of the left ear and auditory tube (1 patient, WAO Grade 2). No TEAEs of WAO Grade 3 or higher were reported. In 36 patients (37.9%), the TEAEs were suspected to be related to IMP (Table 3) and in 6 patients (6.3%) were considered to be related to trial procedures (all related to ACC procedures). Overall, 3 patients experienced 7 serious TEAEs: 1 event (multiple sclerosis) in the grass AIT and 6 events (concussion, road traffic accident, tooth fracture, loss of consciousness, skin erosion, and ventricular extrasystoles) in the birch AIT, which led to discontinuation of 2 patients (multiple sclerosis and ventricular extrasystoles). No serious TEAEs were considered to be related to IMP or procedures.

**Table 3:** Overview of treatment‐emergent adverse events (safety set)

	Grass AIT (N = 47)		Birch AIT (N = 48)		Overall (N = 95)	
	Events	n (%)	Events	n (%)	Events	n (%)
All AEs	108	37 (78.7%)	122	33 (68.8%)	230	70 (73.7%)
AEs related to IMP	60	21 (44.7%)	36	15 (31.3%)	96	36 (37.9%)
Systemic reactions	2	2 (4.3%)	1	1 (2.1%)	3	3 (3.2%)
Local reactions	57	20 (42.6%)	35	15 (31.3%)	92	35 (36.8%)
AEs leading to discontinuation	1	1 (2.1%)	1	1 (2.1%)	2	2 (2.1%)
AEs leading to dose reduction	18	9 (19.1%)	3	1 (2.1%)	21	10 (10.5%)
SAEs	1	1 (2.1%)	6	2 (4.2%)	7	3 (3.2%)
SAEs related to IMP	0	0	0	0	0	0

Abbreviations: AEs, adverse events; AIT, allergen immunotherapy; IMP, Investigational Medicinal Product; N, number of patients; n, number of patients with data; SAE, serious adverse event.

## 4 DISCUSSION

Primary endpoint results demonstrated a treatment effect for grass and birch AIT‐targeted treatments. As previously reported,^8^, ^9^ both allergoid preparations were well tolerated, reduced the allergic rhinoconjunctivitis symptoms, and induced an elevation of allergen‐specific IgG_4_.

Additional key objectives of this trial were to compare treatment effects in the targeted and untargeted AITs, and testing the concept of an “active allergen placebo” vs a conventional placebo. To this end, we compared the targeted treatment effect for grass and birch AIT to the untargeted treatment effect: between targeted treatment groups (ie, between the same allergens for ACC challenges in different AIT groups), within targeted treatment groups (ie, between different allergens for ACC challenges in the same AIT group), and combined (ie, both targeted treatments vs both untargeted).

When comparing targeted vs untargeted treatment effects (AUC_adj_ TNSS) between AIT groups, which evaluates the utility of an “active allergen placebo,” significant differences were observed for both allergens. On within‐treatment group comparisons, which evaluate the specificity of the ACC measurement, the specificity of AIT was only observed for the grass AIT group (G‐G vs B‐G), while for the birch AIT group the difference in treatment effect (B‐B vs G‐B) was not statistically significant. This could be explained by the different level of TNSS response to grass vs birch allergens: Higher levels in response to grass allergen induce a more pronounced separation when comparing targeted (grass, G‐G) to untargeted (birch, B‐G) challenges, while lower levels in response to birch allergen result in less pronounced differences when comparing targeted (birch, B‐B) to untargeted (grass, G‐B) challenges. The combined effect of the targeted treatment, which averages grass and birch treatment effects, was statistically superior to the combined effect of the untargeted treatment. This finding suggests that a stratified assessment according to the magnitude of the allergen TNSS response might be useful to evaluate the ACC measurement specificity. Immunological results further confirmed AIT specificity, in which a significant elevation in specific IgG_4_ allergen was only observed in patients in the targeted AIT. The severity of allergy symptoms during each pollen season (NRS scores), although presenting a similar trend for AIT specificity, did not show statistically significant differences between targeted and untargeted effects for both AIT groups.

Pfaar et al^6^ reported a dose‐finding, placebo‐controlled trial using the same grass IMP, same ACC (Fraunhofer‐ITEM, Hannover) and ACC exposure (120 minutes) with similar challenge agents and pollen concentrations. The treatment duration (October 2012‐January 2013) was, however, shorter (≈4 months) than in our study (≈9 months) and did not include a grass pollen season. Pfaar et al reported a median (25th, 75th) treatment effect based on AUC_adj_ TNSS of ‐6.0 (−14.0, −2.0) for grass AIT and −1.0 (−11.0, 4.0) for placebo. In contrast, we observed a median treatment effect of −12.5 (−23.0, −7.0) in targeted grass AIT and of −5.0 (−10.0, 3.0) and −6.0 (−15.0, 4.0) in untargeted treatments (Table 2). Although the magnitude of the treatment response for grass AIT is higher in our study, the difference between active vs placebo in Pfaar et al, and targeted vs untargeted in our study is comparable. The shorter duration of treatment and the absence of a pollen season during treatment may have caused lower treatment effects in Pfaar et al study. The parallelism between study results is further confirmed by a comparable lack of IgG_4_ increase in untargeted treatments (our study) and in the placebo group (Pfaar et al).^6^

In the early 1980s, Dreborg et al performed a double‐blind, placebo‐controlled trial with a design comparable to our study with mite and grass allergens. The study missed the primary outcome and was only published 30 years later because post hoc analyzed results, confirming the specificity of AIT, made it possible to use an alternate AIT as “active allergen placebo.”^3^ The post hoc analysis was triggered by the observation that the lower side effects of injected histamine placebo compromised the blinding of the study.^3^ Dreborg et al^3^ concluded that AIT is allergen‐specific, as judged by decreased conjunctival sensitivity and changes in allergen‐specific IgG concentrations. We used the same approach in our study; however, we also evaluated symptoms due to seasonal allergens with overlapping pollination in an ACC. As underlined by a 2017 position paper by the European Academy of Allergy and Clinical Immunology,^10^ the use of an ACC facilitates control and standardization of the quantitative allergen exposure. Further advantages are using fewer patients than natural exposure studies, avoiding the variability of pollen seasons and confounding effects of rescue medications,^11^ and easing the dose‐finding process, the verification of onset of action, and the long‐term treatment effects.^12^, ^13^, ^14^

Our study corroborates the ARIA‐GA^2^LEN statement concept that in dual‐allergic patients, with randomization to 1 of 2 active AITs, the respective alternate active treatment group can serve as an “active allergen placebo.” The use of an “active allergen placebo”^1^ enables all the patients to receive active treatments, therefore addressing important ethical concerns: This last point is especially relevant for pediatric studies requested by the European Medicine Agency^5^ since the use of placebo treatment in children is not ethical. As all patients receive active treatment, this trial design may allow longer trial durations, enabling the study of long‐term effects of AIT. Furthermore, this design favors the study blinding since both treatment groups are likely to experience classic AIT AEs.

Although the use of an “active allergen placebo” is an attractive concept supported by our data, interpretation on specificity is limited and would have been more robust with the introduction of an additional classical placebo group. While this would have allowed to assess the specificity and sensitivity of ACC challenges, blinding problems with the classical placebo would have required a larger sample size for robust conclusions. The observed differences in specificity in grass‐ vs birch‐AIT warrant more detailed studies, including stratified assessments according to response levels, to further establish the usability of an “active allergen placebo.” Coupling molecular allergy testing with this study design might better define the study population sensitization profiles (including cross‐reactions) and enhance the evaluation of clinical responses. Another limitation to be considered in this study design is the potential high number of patients that need to be screened to find suitable dual‐allergic patients.

In conclusion, for patients suffering from dual allergies from distinct allergens, the active untargeted treatment is an adequate control group for the active targeted treatment. This finding has implications for future clinical trials as this trial design enables all patients to benefit from treatment, avoiding ethical concerns, improving blinding, and allowing longer trial durations.

## CONFLICTS OF INTEREST

Martin Wagenmann’s institution received funding for the study conduct. He received honoraria for advisory board activities and lectures outside this work from ALK‐Abelló, Allergopharma, AstraZeneca, HAL Allergy, MEDA Pharma, Sanofi‐Aventis, Stallergenes, and Teva outside the submitted work. Margitta Worm is an employee of Charité‐Universitätsmedizin Berlin. She received honoraria as consultant in advisory boards and research grants by ALK‐Abelló Arzneimittel GmbH, Meda Pharma GmbH & Co. KG, Allergopharma GmbH & Co. KG, Bencard Allergie GmbH, Sanofi‐Aventis Deutschland GmbH, and Regeneron Pharmaceuticals Inc. Yasemin Akboga is an employee of Allergopharma GmbH & Co. KG, A business of Merck. Martin Karjalainen is an employee of Allergopharma GmbH & Co. KG, A business of Merck. Jens M. Hohlfeld’s institution received funding for the study conduct and owns an allergen challenge chamber. Jens M. Hohlfeld received honoraria for advisory board activities and lectures outside this work from Boehringer Ingelheim, Leti, MSD, and Novartis.
