# Supplementary material for: Randomized immunotherapy trial in dual‐allergic patients using “active allergen placebo” as control
Source: Allergy. 2019 May 28;74(8):1480–9. doi: 10.1111/all.13842 (PMC6773203; doi:10.1111/all.13842)
Supplement: Supplementary file 1 [file ALL-74-1480-s001.pdf]

Supporting information

Allergen challenge chamber (ACC) technical details 2

Skin prick test (SPT) technical details 3

Immunological profile........ 3

Peak expiratory flow (safety outcome) 4

Table S1 – Peak expiratory flow for patients with controlled asthma (N=27, safety set)........ 4

Table S2 – Peak expiratory flow for all patients (N=95, safety set)........ 4

Change in birch/grass IgG_4........ 5

Figure S1 – Box-plots of targeted and untargeted effects of allergen immunotherapy on specific IgG_4 values (full analysis set) 5

Figure S1a – Individual patient's values of grass/birch IgG_4 in grass AIT group 6

Figure S1b – Individual patient's values of grass/birch IgG_4 in birch AIT group 7

Targeted vs untargeted treatment effects (individual values)........ 8

Figure S2a – Individual patient's total nasal symptom scores in grass AIT group........ 8

Figure S2b – Individual patient's total nasal symptom scores in birch AIT group........ 9

Figure S2c – Treatment effect between-groups in individual patient's values........ 10

Figure S2d – Treatment effect within-groups in individual patient's values 11

Allergen challenge chamber (ACC) technical details

The grass pollen atmosphere was created in the validated ACC according to standardized operating procedures of the Fraunhofer-ITEM (Hannover, Germany). The airflow through the chamber was 1,500 m^3/h. The chamber was charged with indoor air, which was cleaned, temperature-controlled, dried, and then loaded with a qualitatively and quantitatively determined allergen load. Allergen concentration, temperature, and humidity were continuously monitored, allowing for a constant humidity (40% [±10%]), temperature (21°C [±2°C]), and a predefined allergen load (expressed as grains per cubic meter) to be maintained throughout the exposure period of 2 hours. The pollen concentration in the ACC (4,000 grains per cubic meter ± 30%) was continuously measured via a particle counter (Boulder Counter) and discontinuously assessed by taking 2 samples (1 sample after half of the challenge duration, and 1 sample after the challenge was completed) on a pollen sampler (ROTOROD) followed by counting the pollen under the microscope.

Skin prick test (SPT) technical details

A routine SPT was carried out to allow an objective assessment of the sensitization of the patients. SPT was performed using test solutions provided by the sponsor, even if a recent SPT was available for the patient. Concomitant medication had to be checked for interference with SPT and discontinued prior to its conduct. If the Screening Visit took place during a pollen season (birch or grass), the SPT had to be postponed if the patient showed severe acute allergic symptoms. In this case, the optional visit was to be scheduled at a later time point, when the patient was free of symptoms.

For the assessment of the SPT results the following rules were considered:

- For test validity, the positive control was acceptable if the wheal size was ≥3 mm.
- For the negative control, the skin reaction was judged as being negative if the wheal was ≤2mm.
- For the different allergen test solutions, the result was considered to be positive if the wheal size was ≥3 mm.

The SPT was performed with the following test solutions (Allergopharma GmbH & Co. KG, Reinbek / Hamburg, Germany):

- Negative control: physiological saline solution (0.9% NaCl).
- Positive control: 0.1% histamine dihydrochloride (histamine 1 + 999, histamine dihydrochloride 1.7 mg/mL).
- Birch (108 Birch; 50,000 SBU [standardized biological units]/mL).
- Allergen extracts from a mixture of 6 grasses (006 Grasses: 133 *Holcus lanatus*, 140 *Dactylis glomerata*, 157 *Lolium perenne*, 177 *Phleum pratense*, 178 *Poa pratensis*, and 179 *Festuca pratensis*; 50,000 SBU/mL).
- Alder (115 Alder; 50,000 BU [biological units]/mL).
- Hazel (129 Hazel; 50,000 BU/mL).
- House dust mites (708 *Dermatophagoides [D.] farinae*; 50,000 BU/mL).
- House dust mites (725 *D. pteronyssinus*; 50,000 SBU/mL).

Immunological profile

At Screening Visit, the immunological profile was assessed, including the following parameters:

- Total immunoglobulin E (IgE).
- Specific IgE Grass mix, IgE Birch, IgE Ragweed, IgE Mugwort, IgE Plantain, IgE Alder, IgE Hazel, IgE *D. pteronyssinus d1*, IgE *D. farinae d2*, IgE Dog, and IgE Cat.
- Specific immunoglobulin G_4 (IgG_4) timothy grass and IgG_4 birch.

At Final Visit:

- Specific IgG_4 timothy grass and IgG_4 birch.

Peak expiratory flow (safety outcome)

Table S1 – Peak expiratory flow for patients with controlled asthma (N=27, safety set)

**Patients with controlled asthma (GINA, 2012)**		**Grass AIT (N=10)**		**Birch AIT (N=17)**		**Overall (N=27)**	
		PEF (L/min)	Predicted PEF (%) [a]	PEF (L/min)	Predicted PEF (%) [a]	PEF (L/min)	Predicted PEF (%) [a]
**Baseline**	n	10	10	17	17	27	27
	Mean (SD)	525 (120)	102 (10)	490 (125)	98 (12)	503 (122)	99 (11)
	95% CI	439 – 611	94 – 109	425 – 554	92 – 104	454 – 551	95 – 104
**Final Visit**	n	10	10	17	17	27	27
	Mean (SD)	518 (94)	101 (12)	481 (137)	96 (12)	495 (122)	98 (12)
	95% CI	451 – 585	93 – 110	411 – 552	90 – 102	446 – 543	93 – 103
**Final Visit - Baseline**	n	10	10	17	17	27	27
	Mean (SD)	-7.0 (46.7)	-0.1 (8.9)	-8.4 (57.1)	-1.9 (10.3)	-7.9 (52.6)	-1.2 (9.7)
	95% CI	-40.4 – 26.4	-6.5 – 6.3	-37.7 – 21.0	-7.1 – 3.4	-28.7 – 12.9	-5.0 – 2.6

[a] Percentage of predicted value according to European Community for Steel and Coal

AIT = allergen immunotherapy; CI = confidence interval; GINA = Global Initiative for Asthma; N = number of patients; n = number of patients with data; PEF = peak expiratory flow; SD = standard deviation.

Table S2 – Peak expiratory flow for all patients (N=95, safety set)

**All Patients**		**Grass AIT (N=47)**		**Birch AIT (N=48)**		**Overall (N=95)**	
		PEF (L/min)	Predicted PEF (%) [a]	PEF (L/min)	Predicted PEF (%) [a]	PEF (L/min)	Predicted PEF (%) [a]
**Baseline**	n	47	47	48	48	95	95
	Mean (SD)	514 (107)	102 (11)	499 (120)	101 (11)	507 (113)	102 (11)
	95% CI	483 – 546	99 – 106	464 – 534	98 – 104	484 – 530	100 – 104
**Final Visit**	n	28	28	34	34	62	62
	Mean (SD)	493 (97)	98 (11)	498 (127)	102 (13)	495 (113)	100 (12)
	95% CI	455 – 530	94 – 103	453 – 542	97 – 106	467 – 524	97 – 103
**Final Visit - Baseline**	n	28	28	34	34	62	62
	Mean (SD)	-10.4 (36.7)	-1.5 (6.9)	6.1 (56.6)	1.6 (11.6)	-1.3 (49.0)	0.2 (9.8)
	95% CI	-24.6 – 3.9	-4.1 – 1.2	-13.6 – 25.9	-2.5 – 5.6	-13.8 – 11.1	-2.3 – 2.7

[a] Percentage of predicted value according to European Community for Steel and Coal

AIT = allergen immunotherapy; CI = confidence interval; N = number of patients; n = number of patients with data; PEF = peak expiratory flow; SD = standard deviation.

Change in birch/grass IgG4

Figure S1 – Box-plots of targeted and untargeted effects of allergen immunotherapy on specific IgG4 values (full analysis set)

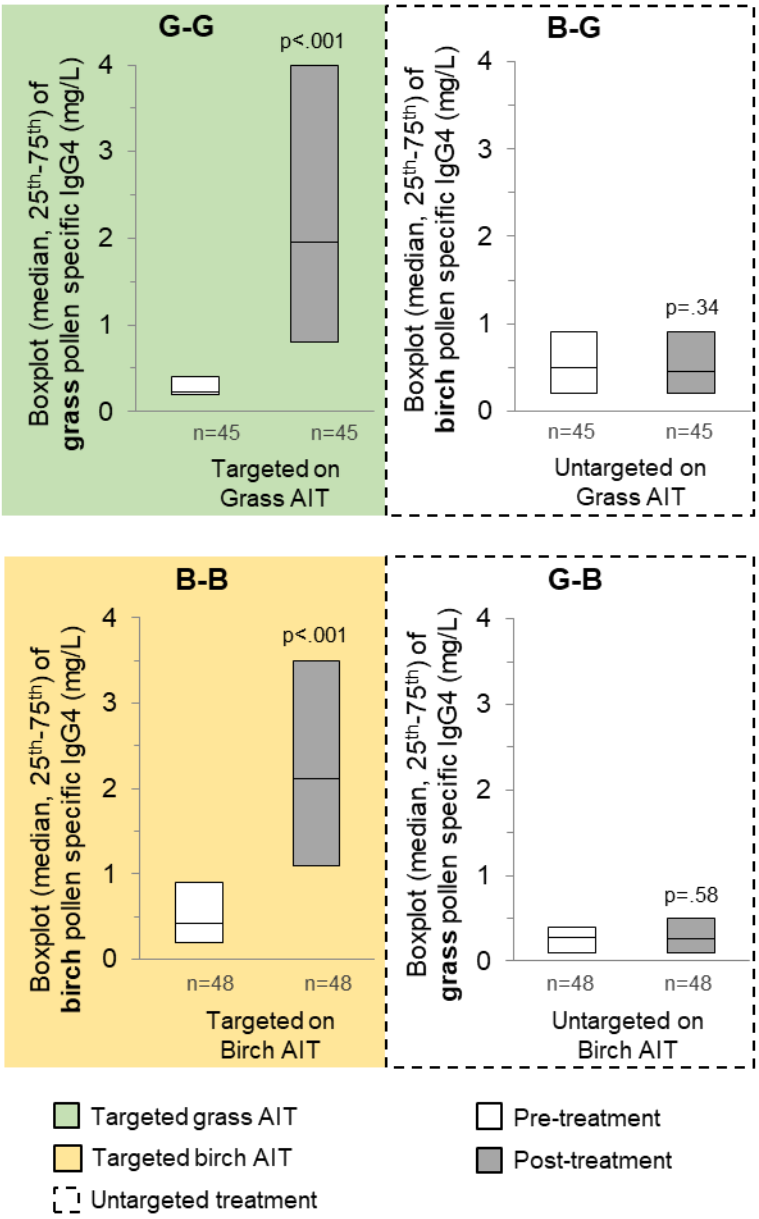

p-value from 2-sided Wilcoxon signed rank test.

Results for the per-protocol set analysis were similar to full analysis set.

AIT = allergen immunotherapy; **B-B** = birch-on-birch treatment effect (targeted); **B-G** = birch-on-grass treatment effect (untargeted); **G-B** = grass-on-birch treatment effect (untargeted); **G-G** = grass-on-grass treatment effect (targeted); IgG4 = immunoglobulin G4; n = number of patients with data.

Figure S1a – Individual patient's values of grass/birch IgG4 in grass AIT group

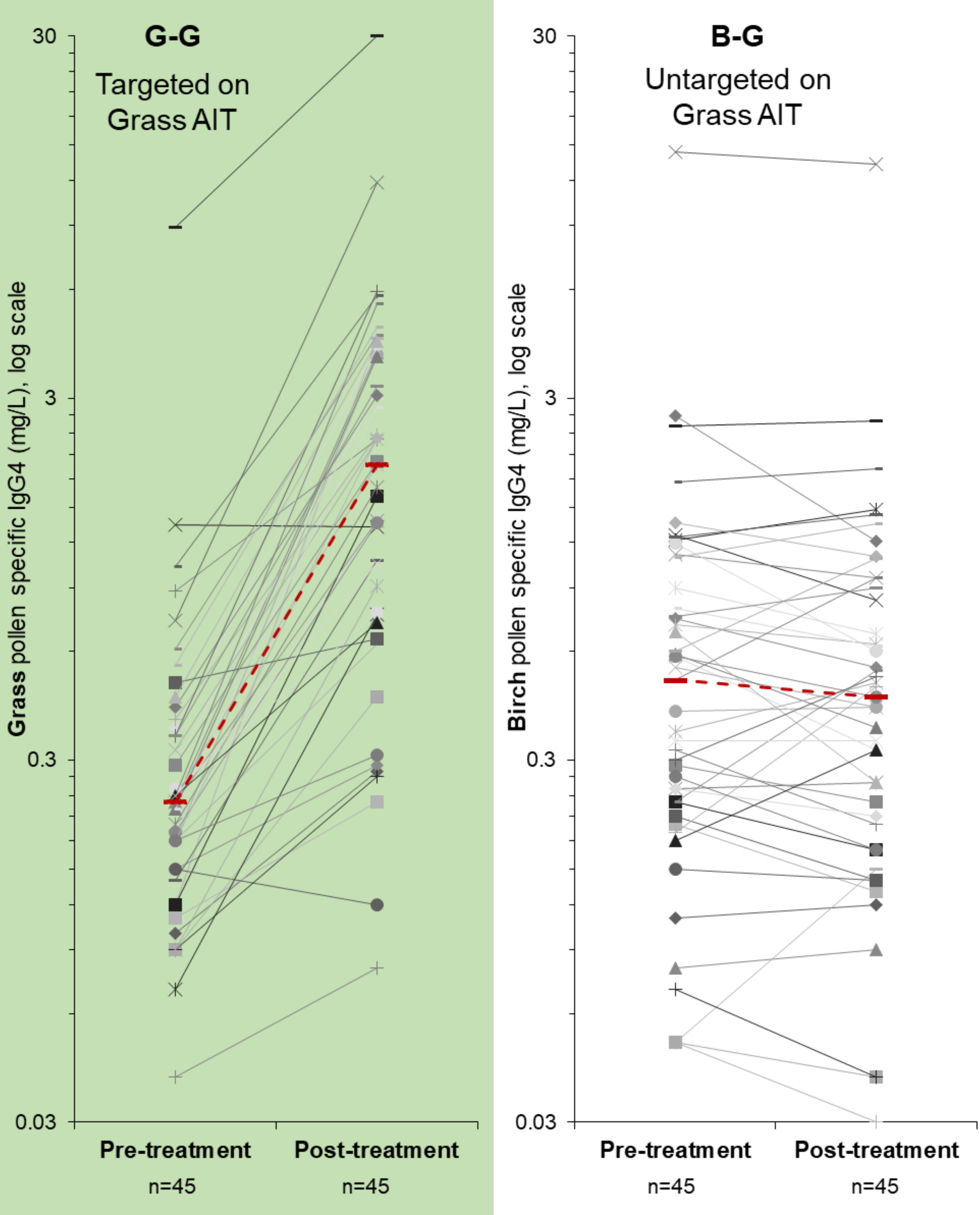

Pre- and post-treatment values are connected by a line for each patient.

Red markers and red dashed line represent median values.

AIT = allergen immunotherapy; **B-G** = birch-on-grass treatment effect; **G-G** = grass-on-grass treatment effect; IgG_4 = immunoglobulin G_4; n = number of patients with data.

Figure S1b – Individual patient's values of grass/birch IgG_4 in birch AIT group

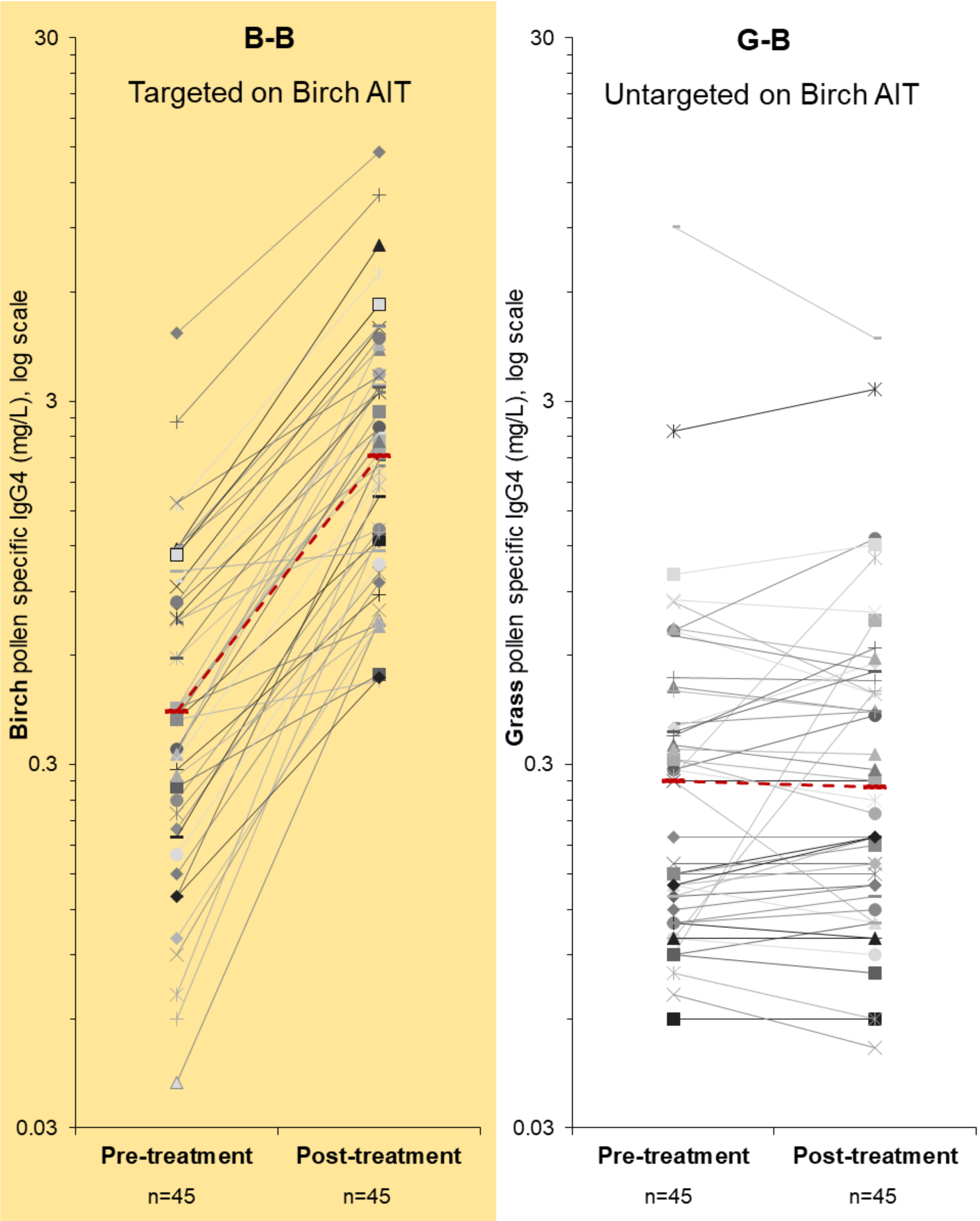

Pre- and post-treatment values are connected by a line for each patient.

Red markers and red dashed line represent median values.

AIT = allergen immunotherapy; **B-B** = birch-on-birch treatment effect; **G-B** = grass-on-birch treatment effect; IgG_4 = immunoglobulin G_4; n = number of patients with data.

Targeted vs untargeted treatment effects (individual values)

Figure S2a – Individual patient's total nasal symptom scores in grass AIT group

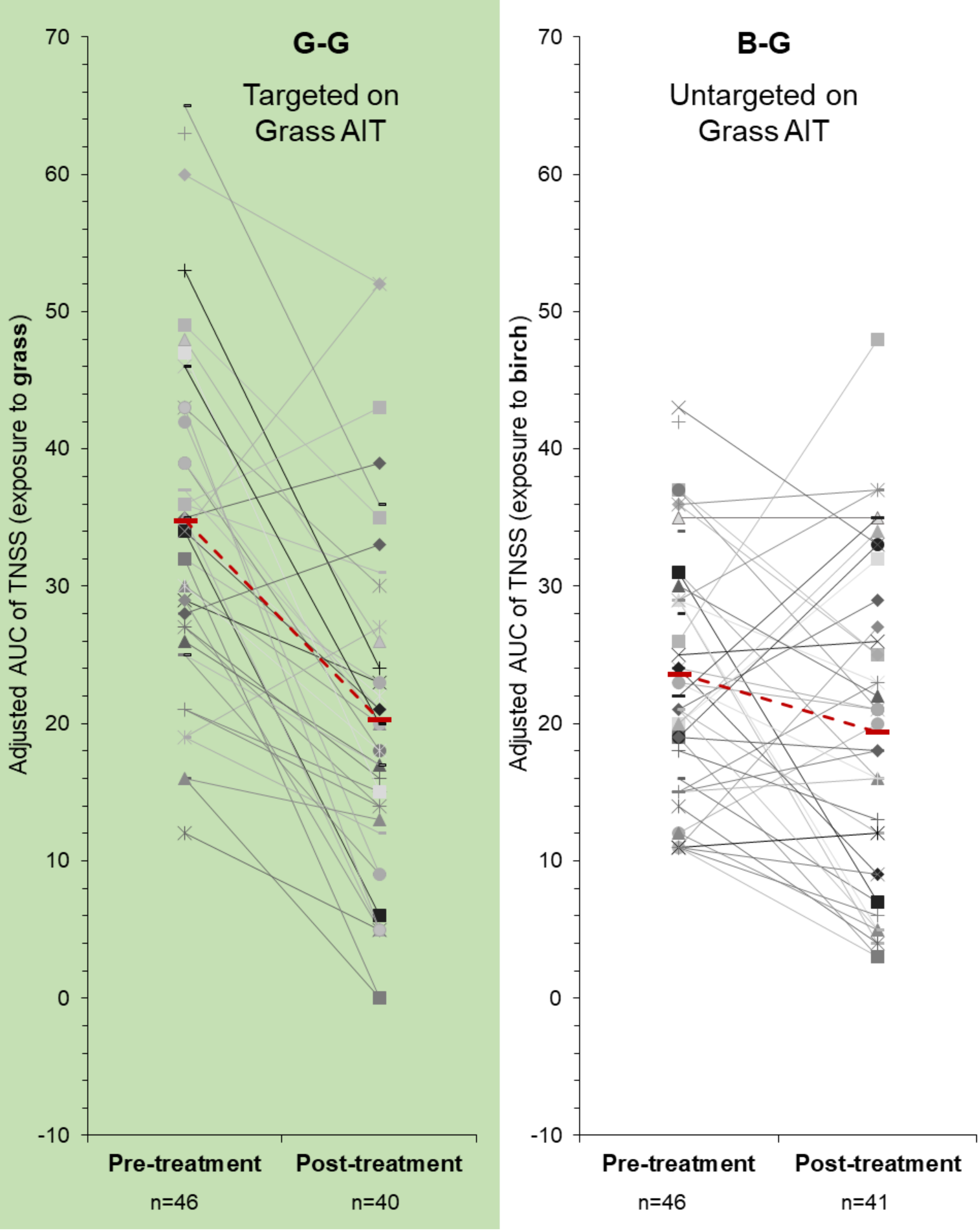

Pre- and post-treatment values are connected by a line for each patient.

Red markers and red dashed line represent mean values.

AIT = allergen immunotherapy; AUC = area under the curve; **B-G** = birch-on-grass treatment effect; **G-G** = grass-on-grass treatment effect; n = number of patients with data; TNSS = total nasal symptom score.

Figure S2b – Individual patient's total nasal symptom scores in birch AIT group

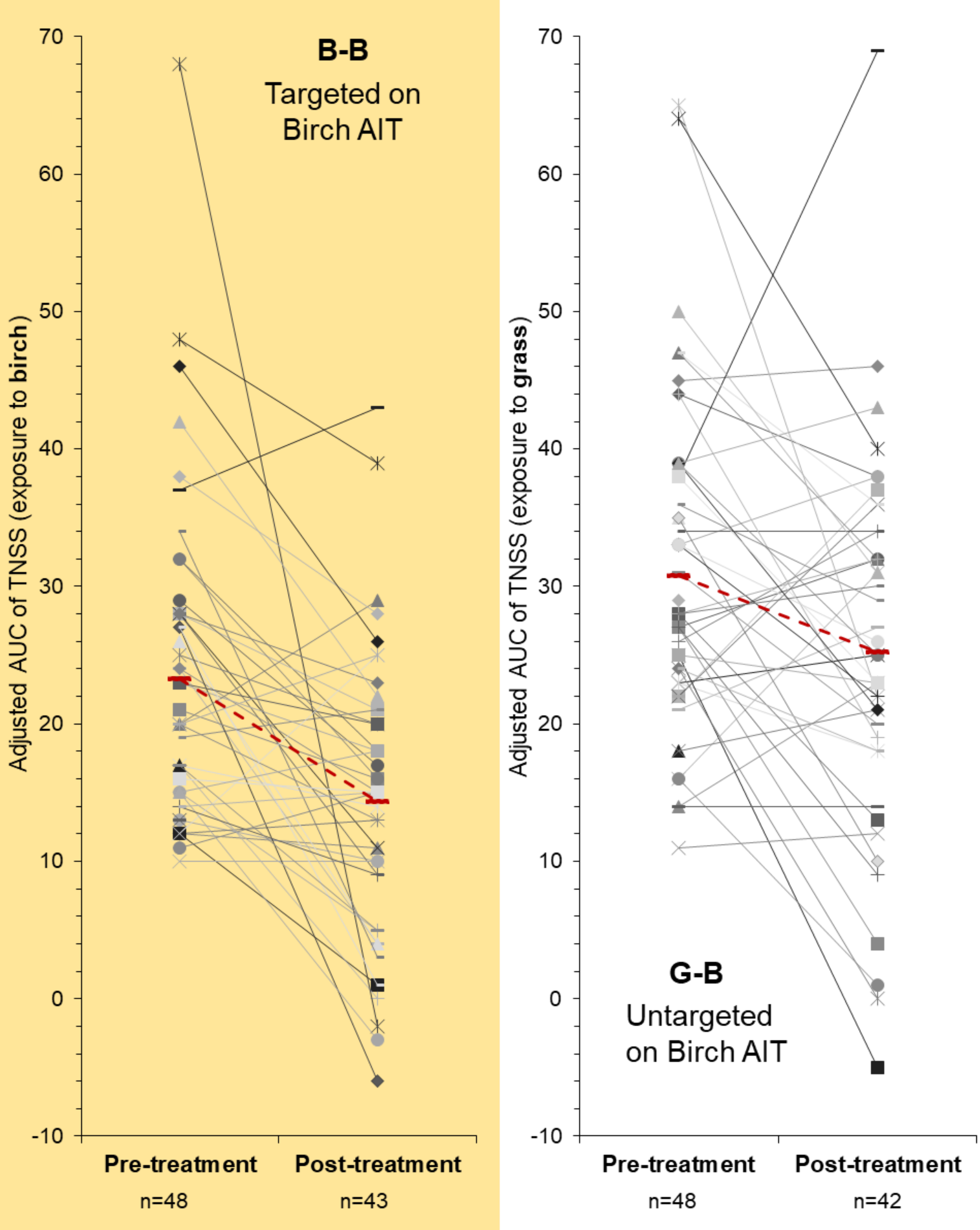

Pre- and post-treatment values are connected by a line for each patient.

Red markers and red dashed line represent mean values.

AIT = allergen immunotherapy; AUC = area under the curve; **B-B** = birch-on-birch treatment effect; **G-B** = grass-on-birch; IgG_4 = immunoglobulin G_4; n = number of patients; TNSS = total nasal symptom score.

Figure S2c – Treatment effect between-groups in individual patient's values

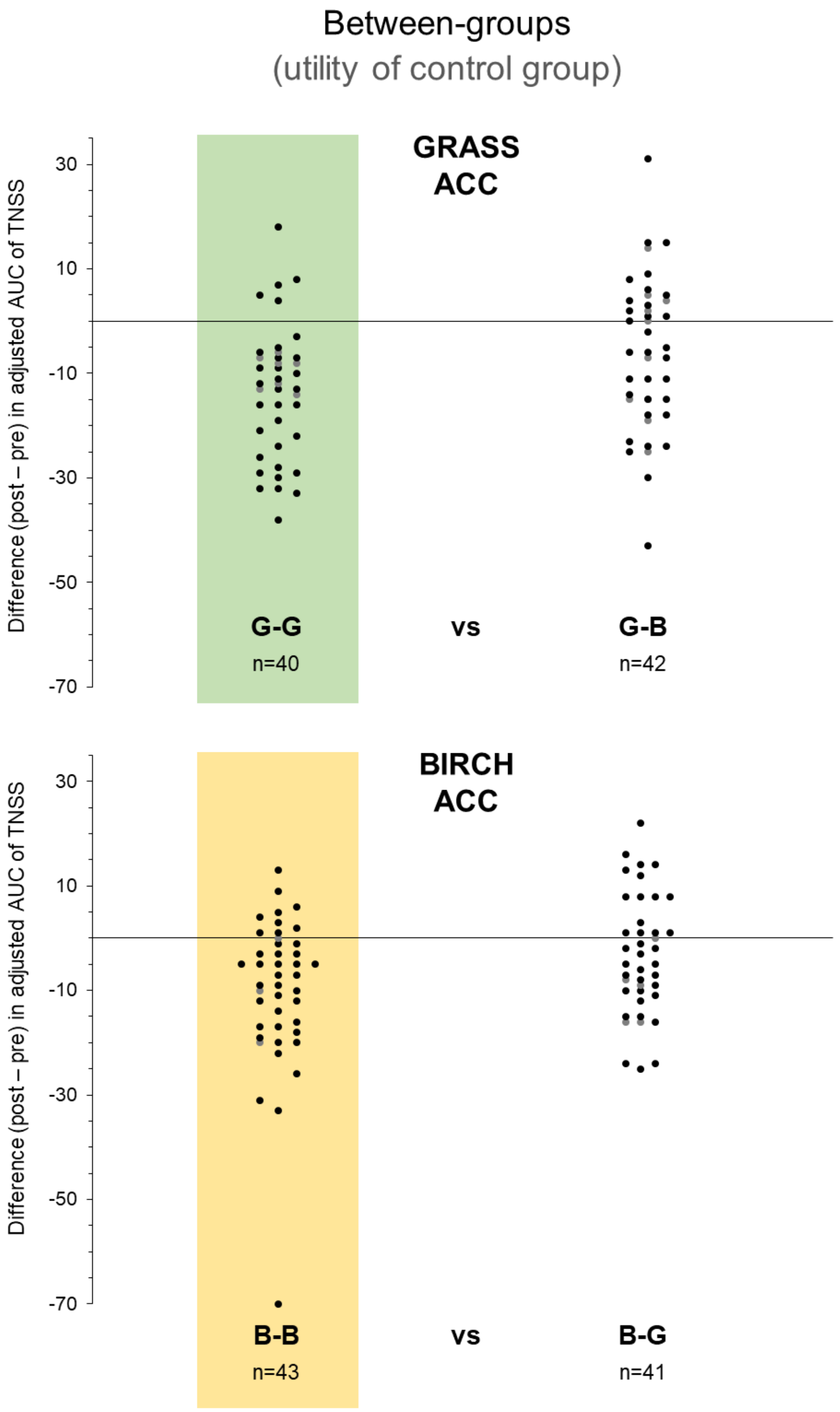

Dots (black mostly/grey when overlapping) represent individual patient values.
AUC = area under the curve; **B-B** = birch-on-birch treatment effect (targeted); **B-G**= birch-on-grass treatment effect (untargeted); **G-B** = grass-on-birch treatment effect (untargeted);
G-G = grass-on-grass treatment effect (targeted); n = number of patients with data; TNSS = total nasal symptom score.

Figure S2d – Treatment effect within-groups in individual patient's values

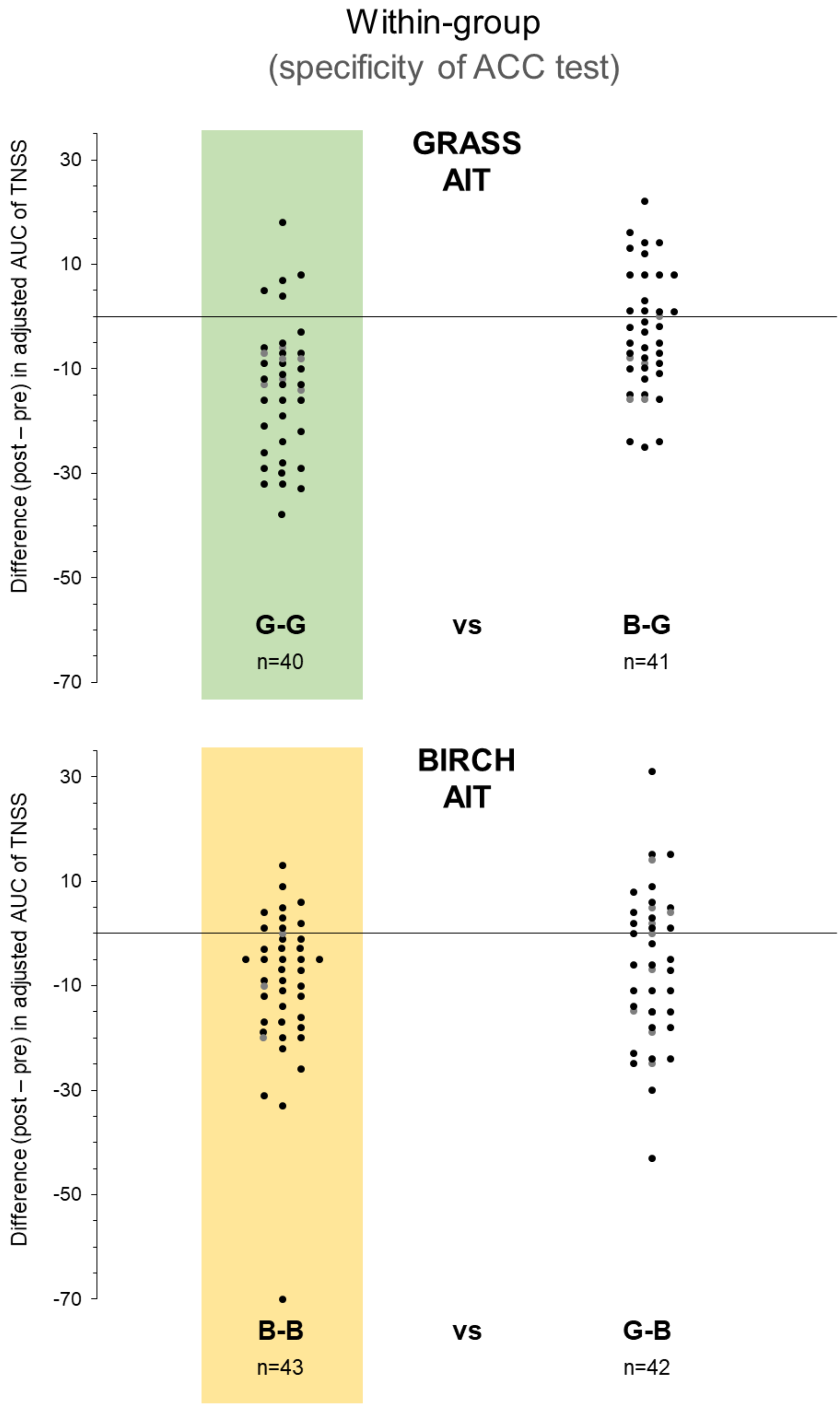

Dots (black mostly/grey when overlapping) represent individual patient values.
ACC = allergen challenge chamber; AIT = allergen immunotherapy; AUC = area under the curve; **B-B** = birch-on-birch treatment effect (targeted); **B-G** = birch-on-grass treatment effect (untargeted); **G-B** = grass-on-birch treatment effect (untargeted); **G-G** = grass-on-grass treatment effect (targeted); n = number of patients with data.
